# Rapid, direct, and sensitive determination of aziridine and 2-chloroethylamine by hydrophilic interaction liquid chromatography-mass spectrometry

**DOI:** 10.1016/j.mex.2019.09.017

**Published:** 2019-09-18

**Authors:** Jonathan G. Shackman

**Affiliations:** Chemical and Synthetic Development, Bristol-Myers Squibb Company, 1 Squibb Drive, New Brunswick, NJ, 08901, USA

**Keywords:** HILIC-MS detection of aziridine and 2-chloroethylamine, Aziridine, 2-Chloroethylamine, Genotoxic impurity, HILIC-MS

## Abstract

A rapid HILIC-MS method was developed for measuring the genotoxic impurities aziridine and 2-chloroethylamine. Sample preparation was simple and direct without requiring derivatization. Paired with a 1.5 min isocratic UHPLC separation, sample analysis could be completed in less than 5 min. Linearity was established from 0.5 μg/L to 10 μg/L for both target analytes. For main components at 100 g/L, this equates to 5 parts per billion (ppb) detectability using a benchtop, single quadrupole detector. Three model matrices were evaluated (glycine, phenylalanine, and the pharmaceutical drug asunaprevir), and the method was able to provide suitable repeatability (<10% RSD) and accuracy (±10%) at 5 μg/L concentrations.

• Direct sample preparation without derivatization as is needed for GC analyses
• Less than 5 min required for sample preparation and HILIC-MS analysis
• Part per billion sensitivity in multiple test matrices with good recovery

## Graphical abstract

**Figure:**
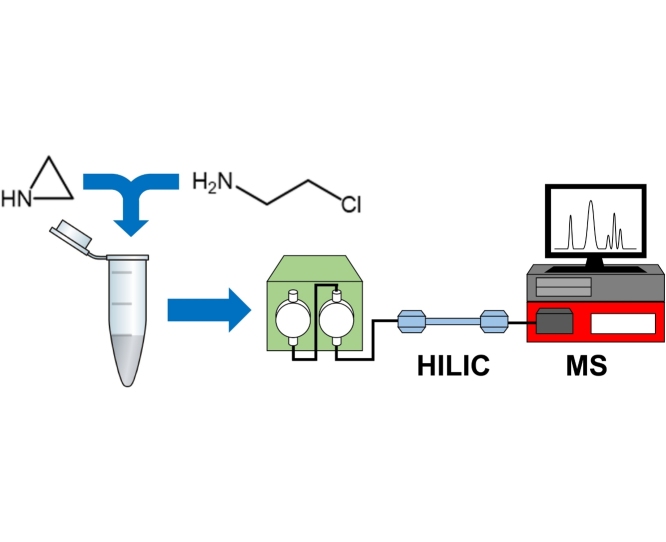

Specification Table

**Table:** 

Subject Area:	Chemistry
More specific subject area:	Analytical Chemistry
Method name:	HILIC-MS detection of aziridine and 2-chloroethylamine
Name and reference of original method:	N/A
Resource availability:	All resources commonly available

## Method details

### Background

**Fig. 1:**
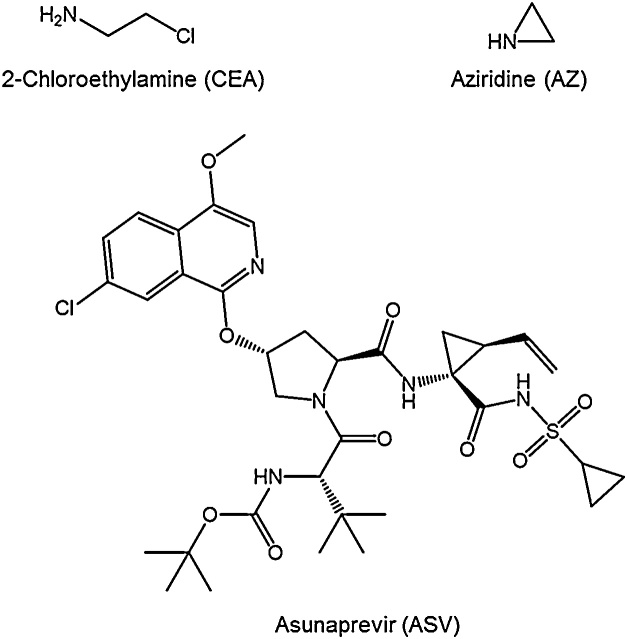
Analyte and drug compound structures.

Alkylamines, such as 2-chloroethylamine (CEA; Fig. 1), are common synthetic chemistry reagents. CEA can also undergo self-cyclization, forming aziridine (AZ; Fig. 1) [1], a highly reactive and mutagenic compound [2]. Both CEA and AZ must be controlled in pharmaceuticals following the International Conference on Harmonization (ICH) M7 guidelines [3]. Although the required analytical sensitivity for assessing these components in pharmaceutical intermediates, active substances, or products will be dictated by the dosage amount and duration, typically low parts per million (ppm; μg/g) limits of detection (LOD) are needed. Previous reports of AZ and / or CEA analysis combined with a separation technique (*e.g.,* LC or GC) required manual derivatization and extraction in order to obtain low level detection from a concentrated main component matrix [[4], [5], [6], [7], [8]].

Ideally, direct analysis of AZ and CEA would be preferred in order to improve the method’s simplicity and robustness by removing the possibility of incomplete or inconsistent derivatization and / or extraction. Recently, Douša et al. described a method using hydrophilic interaction liquid chromatography coupled with mass spectrometry detection (HILIC–MS) for measuring 2-chloro-*N*-(2-chloroethyl) ethanamine in vortioxetine using an HPLC mixed mode column (Primesep B, SIELC Technologies), making it unclear if the dominant retention mechanism was HILIC partitioning or ion exchange [9]. No derivatization or extraction was necessary, LC analysis required ∼10 min, and a 0.3 μg/L LOD was reported, allowing for measurements to 300 ppb in vortioxetine. The present work adapts the HILIC-MS approach for trace level determinations of AZ and CEA. For direct comparison to sample derivatization with GC–MS analysis as described in [8], glycine (GLY) and phenylalanine (PHE) matrices were tested, as well as asunaprevir (ASV; Fig. 1) as a model pharmaceutical active substance.

### Chemicals and sample preparation

AZ (1 g/L in methanol) was from SPEX CertiPrep (Metuchen, NJ, USA). 99% CEA, HPLC gradient grade acetonitrile, > 99.995% ammonium formate, ACS reagent grade GLY, and >99% L-phenylalanine (PHE) were from Sigma-Aldrich (St. Louis, MO, USA). Water was purified in-house using an Advantage A10 purification system (Millipore Sigma, Burlington, MA, USA). ASV potassium salt was from Bristol-Myers Squibb (New Brunswick, NJ, USA). All samples and standards were prepared in an ammonium formate (0.1 M) : acetonitrile (5 : 95, v/v) diluent; pH was unadjusted. All standards had both analytes of interest (AZ and CEA) present together. Samples were directly weighed into 1.7 mL polypropylene centrifuge tubes (VWR, Radnor, PA, USA), diluted to either 50 or 100 g/L by autopipette, vortexed for 30 s (Vortex Genie 2, Thermo Fisher Scientific, Inc., Waltham, MA, USA) to a uniform solution or slurry, and centrifuged at 14,000 RPM for 1 min (5415C, Eppendorf, Hauppauge, NY, USA). The supernatant (if undissolved material remained) was transferred to an HPLC vial for analysis.

### Instrumentation and chromatographic conditions

HILIC-MS used a Waters Acquity HClass UPLC with an SQD detector. Mobile phase was ammonium formate (0.1 M) : acetonitrile (5 : 95, v/v); pH was unadjusted. 10 μL of sample or standard maintained at 10 °C were injected on a Waters BEH HILIC 50 mm × 2.1 mm, 1.7 μm particle size, column maintained at 30 °C with a flow rate of 0.5 mL/min. Flow was directed to waste from 0.0 to 1.0 min and into the MS from 1.0 to 1.5 min *via* divert valve. Selected ion monitoring (SIM) using positive electrospray ionization was performed at 44.1 (AZ) and 79.9 (CEA) Da. Tuning parameters were: source temperature of 150 °C; desolvation temperature of 450 °C; desolvation gas flow of 900 L/h; capillary at 3 kV, cone at 30 V, and extractor at 3 V.

### Method performance

As the recently described GC derivatization methodology developed by Zapata et al. [8] exhibited excellent analytical figures of merit, a similar approach was used for evaluating the HILIC-MS technique so that comparisons can be made. Specifically, GLY and PHE were used as surrogate hydrophilic and hydrophobic main components, respectively. ASV was a true small-molecule drug substance matrix. A short (5 cm) UHPLC (sub-2-μm particle size) column was selected in order to maximize speed, with the BEH stationary phase preferred due to its general robustness as compared to pure silica or bonded phases [10]. A 1.5 min isocratic run time with a retention factor of ∼4 for the earliest eluting analyte (AZ) was obtained, allowing for adequate resolution from the void volume containing the unretained and overloaded main components. For the benchtop MS, the manufacturer’s suggested default tuning parameters for high eluent flow rates were found to have sufficient sensitivity, with little improvement observed by altering them. No interfering peaks were observed from the three sample matrices at either SIM channel.

**Fig. 2:**
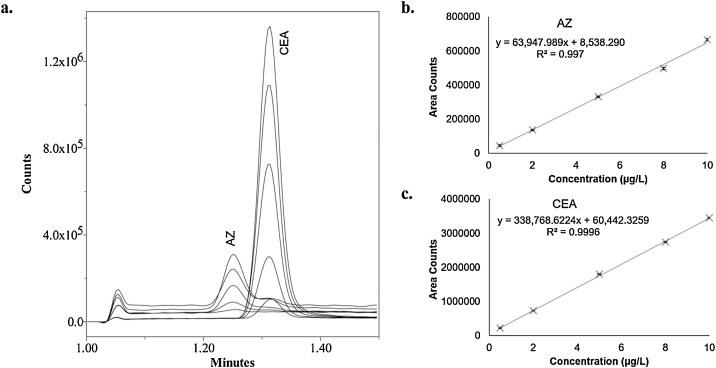
a. Representative SIM chromatograms of 0.5 μg/L to 10 μg/L calibration standards. AZ was monitored at 44.1 *m/z* and CEA was monitored at 79.9 *m/z*. Linearity of b. AZ and c. CEA.

The HILIC-MS method was calibrated using analyte standards from 0.5 μg/L to 10 μg/L (triplicate injections at each level; Fig. 2). R^2^ values greater than 0.99 were obtained. Using an initial sample matrix target concentration of 100 g/L, this would correspond to a linear range from 5 to 100 ppb. Calibration was performed with both analytes in each standard at equal levels, and, at the higher concentrations of CEA, a fragment ion begins to be observed in the AZ SIM channel. However, there was sufficient chromatographic resolution (R_s_ = 1.4) to prevent gross inaccuracy, as evidenced by the linearity. Additionally, at the mid-range target recovery level of 50 ppb there was minimal interference between the analytes. The 0.5 μg/L standards had an average S/N of 22 for CEA and 4 for AZ, which are above normally acceptable LOD targets, and injection precision of 3% and 10% relative standard deviation (RSD; n = 6) for CEA and AZ, respectively. Injection precision was improved to <1% RSD for both analytes at concentrations of at least 2 μg/L.

**Fig. 3:**
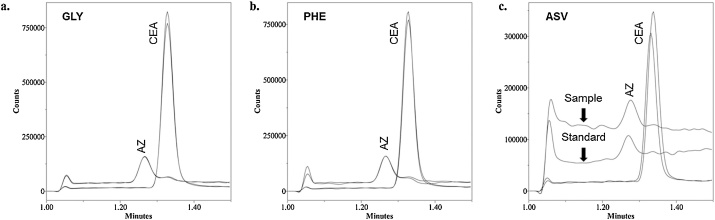
Overlays of representative 50 ppb standard and recovery SIM chromatograms. a. GLY (100 g/L) with 5 μg/L standards. b. PHE (100 g/L) with 5 μg/L standards. c. ASV (50 g/L) with 2.5 μg/L standards. The baseline disturbance at 1.05 min was due to the MS divert valve switching. Only ASV had a notable difference (elevated baseline for AZ SIM) between recovery samples and standards.

Sample preparation for the model compounds was minimal, as they were simply weighed, diluted, vortex mixed to uniformity, and centrifuged to pelletize any insoluble material, if present. All preparation steps can be performed in less than 5 min. The mobile phase was used as diluent to prevent chromatographic perturbations. To demonstrate the HILIC-MS method’s accuracy, spike and recovery (n = 3) at 50 ppb was initially performed with all three model compounds at 100 g/L and the 5 μg/L analyte standards. As shown in Table 1, even at this low quantitation level, GLY and PHE demonstrated excellent sample repeatability (<4% RSD, n = 3) and recovery (within 5%). However, ASV exhibited lower recovery and slightly worse precision. Presumably, this was a matrix effect. Reduction of the working concentration to 50 g/L and subsequent spiking with a 2.5 μg/L (to maintain a 50 ppb limit) improved the precision and reached an acceptable recovery value (within 10%), although higher background noise was still evident in the ASV AZ SIM channel (Fig. 3c). Representative recovery chromatograms for all three matrices are shown in Fig. 3.

**Table 1:** Analytical figures of merit at 50 ppb levels of AZ and CEA in the model matrices (n = 3).

	Repeatability (%RSD)				Recovery (%)			
	GLY (100 g/L)	PHE (100 g/L)	ASV (100 g/L)	ASV (50 g/L)	GLY (100 g/L)	PHE (100 g/L)	ASV (100 g/L)	ASV (50 g/L)
**AZ**	2.8	3.6	3.8	9.9	103	91	84	91
**CEA**	2.3	1.0	10.8	4.2	105	102	86	91
